# Cost-effectiveness of 2NRTI+NNRTI versus 2NRTI+PI as the initial cART regimen

**DOI:** 10.1186/1758-2652-13-S4-P237

**Published:** 2010-11-08

**Authors:** F Aragão, I Vaz Pinto, J Vera

**Affiliations:** 1 National School of Public Health, Avenida Padre Cruz, Lisbon, Portugal; 2 Centro Hospitalar de Cascais, Cascais, Portugal

## Purpose of the study

To compare ART initiation with 2NRTI+NNRTI versus 2NRTI+PI through a retrospective analysis and incorporate these results in a cost-effectiveness analysis.

## Methods

Time to regimen switch (discontinuing at least one ARV) due to VF (two consecutive viral loads >50 cps/ml after viral suppression or 6-months unreached viral suppression), time to regimen switch due to any other reason and time to resistance development were calculated using survival analysis. Log-rank test for equality of survivor functions was used for comparison. Average 4-months absolute increase in CD4 cell count was compared using weighted linear least squares. Cost-effectiveness analysis was performed using a microsimulation discrete events model with a lifetime time horizon and 5% discount rate. All resources valued at 2009 prices. The hospital's perspective was assumed.

## Summary of results

The retrospective analysis was performed on a cohort of 317 HIV-1 infected naïve to ART individuals followed at an HIV Unit in Portugal (Centro Hospitalar de Cascais). All (unrandomized) patients in the analysis initiated ART between 2000 and 2008 with either 2NRTI+NNRTI (158) or 2NRTI+PI (159). Median age was 39 years-old, 33% were women, and 29.7% were HCV co-infected. Median (IQR) CD4 count and log10 viral load were 229 (121—350) cells/mm^3^ and 5.0 (4.3—5.5) log cp/ml. Groups were identical with respect to these characteristics. Equally of Kaplan-Meier survival curves could not be rejected with respect to: time to switch due to VF, time to viral suppression and time to resistance development. In the first 3 years, no statistically significant difference between groups was found in CD4 cell count change. Median (95% CI) time to any ARV switch due to reasons other than VF, was 6.7 (3.5-7.1) and 2.7 (2.3-3.7) years in the NN and PI groups, respectively (p=0.0078). The median difference (95% CI) in first regimen monthly ART costs and in monthly non-ART costs was, respectively, 348€ (287€-410€) and 23€(11€-35€), with lower costs for the NN group. Initiating therapy with 2NRTI+NN reduces the average number of switches by 17%, saves 18,943€ per individual and increases life expectancy by 1.3 months due to the impact of the accumulated number of regimens on future events.

## Conclusions

This study suggests that, when clinically valid, initiating therapy with 2NRTI+NN is a cost-saving strategy and equally effective when compared to 2NRTI+PI as the first regimen.

